# Rosette-forming glioneuronal tumor of the cerebellum with intratumoral hemorrhage: case report with radiologic–pathologic correlation in a resource-limited setting

**DOI:** 10.1007/s00381-026-07284-y

**Published:** 2026-04-28

**Authors:** Raymond Chimatira

**Affiliations:** 1 Division of Anatomical Pathology, University of Cape Town, Anzio Road, Observatory, Cape Town, 7925 South Africa; 2 Department of Anatomical Pathology, Groote Schuur Hospital, National Health Laboratory Service, Western Cape, Cape Town, South Africa

**Keywords:** Rosette-forming glioneuronal tumor, Posterior fossa, Cerebellum, Hemorrhage, Histopathology, Radiologic-pathologic correlation

## Abstract

**Background:**

Rosette-forming glioneuronal tumor (RGNT) is a rare, low-grade glioneuronal neoplasm of the posterior fossa, most commonly affecting children and young adults. Although typically indolent, its variable clinical and radiologic presentation may pose diagnostic and management challenges.

**Case report:**

The present case is that of a 19-year-old woman who presented with headache and vomiting and was found to have a hemorrhagic, multicystic lesion centered on the right cerebellar peduncle. Serial radiological imaging demonstrated lesion persistence and stability over a period of more than 12 months, prompting surgical debulking. Histopathologic examination revealed a biphasic glioneuronal tumor composed of true neuropil-centered neurocytic rosettes within a fibrillary glial background. Immunohistochemistry revealed synaptophysin positivity within the rosette cores, glial fibrillary acidic protein (GFAP) expression confined to the glial component, and a low proliferative index (< 1%), meeting the World Health Organization (WHO) essential diagnostic criteria for RGNT, CNS WHO grade 1. Molecular testing was not performed.

**Conclusion:**

This case illustrates the diagnostic value of integrated clinical, radiologic, and pathologic assessment in establishing a confident diagnosis of RGNT, even in the setting of atypical hemorrhagic presentation. Recognition of classic morphologic and immunophenotypic features remains central to accurate diagnosis and appropriate management, particularly in resource-limited settings where access to molecular testing is limited.

## Introduction

Rosette-forming glioneuronal tumor (RGNT) is a rare mixed glioneuronal neoplasm of the central nervous system (CNS), classified as a CNS World Health Organization (WHO) grade 1 tumor. Diagnosis is based on the identification of characteristic biphasic histomorphologic features demonstrating a neurocytic component consisting of small round neurocytes forming neurocytic rosettes and/or perivascular rosettes, and a glial component consisting of piloid and oligodendroglial-like cells [1]. RGNTs are low-grade neoplasms and generally follow an indolent clinical course, although isolated cases with aggressive behavior have been described [1–3].

## Historical background

Kuchelmeister et al. initially described the neoplasm as a dysembryoplastic neuroepithelial tumor of the cerebellum in 1995 [4–6]. In 2002, Komori et al. renamed the entity “rosette-forming glioneuronal tumor” and described it as a distinct group of low-grade mixed glioneuronal tumors arising in the posterior fossa, primarily located in the fourth ventricle and midline structures of the brain [5–7]. Subsequently, the tumor was classified as a distinct entity in the 4th edition of the WHO classification of CNS tumors (2007) [5, 6]. While initially described as a tumor arising in relation to the fourth ventricle, cases involving other sites of the CNS have also been reported [1, 7, 8]. The aetiopathogenesis of RGNT has not been fully elucidated [2, 5]. However, neuroimaging, histological, and molecular findings suggest that RGNT may arise from brain tissue surrounding the ventricle system, while it is postulated that cases arising in the fourth ventricle arise from pluripotent cells of the subependymal plate (periventricular germinal matrix) [1, 2, 5]. In recent years, molecular studies have revealed that RGNTs exhibit a distinct DNA methylation profile and have identified the presence of *MAPK* pathway mutations, most often associated with *FGFR1* (fibroblast growth factor receptor 1) mutations, as well as the co-occurrence of *PIK3CA* and *NF1* mutations [1, 9].

## Clinical presentation

RGNTs are slow-growing tumors that predominantly affect children, adolescents, and young adults (mean age of onset 26 years; range 4 to 81 years), and have been reported in association with genetic susceptibility syndromes, including neurofibromatosis type 1 and Noonan syndrome [1, 2, 6, 9–11]. There is a slight female predilection, with a female-to-male ratio of approximately 1.4–2:1 [5, 9, 10, 12]; and data on population-specific incidence are not available due to the rarity of the tumors [1, 9]. The tumors most commonly arise in midline posterior fossa structures, particularly the fourth ventricle and cerebellum [1, 2]. Cases involving the pineal gland, spinal cord, optic nerve, septum pellucidum, and the cerebral hemispheres have been described [1, 2, 6, 8, 13, 14].

The typical presentation of RGNT reflects its location in the fourth ventricle or cerebellum, leading to raised intracranial pressure or obstructive hydrocephalus. Patients most commonly report headaches, dizziness, ataxia, dysarthria, diplopia, blurred vision, or vomiting due to raised intracranial pressure or hydrocephalus [1, 3, 5, 6, 10]. Rarely, spinal cord involvement causes sensory deficits or compressive myelopathy [2, 8]. Rare asymptomatic cases discovered as incidental imaging findings have been described [1, 6, 10].

## Diagnosis

Rosette-forming glioneuronal tumors are typically slow growing, with diagnosis often delayed for several years after symptom onset. Accurate diagnosis depends on an integrated evaluation of clinical findings, typical radiologic features that demonstrate stability over time without aggressive features, and definitive histopathologic confirmation [9, 10].

On computerized tomography (CT) imaging, RGNTs appear hypodense, often appearing as a mixed solid-cystic structure. Intratumoral hemorrhage and calcification may be present, and there is minimal to no enhancement on contrast-enhanced CT scan [1, 9, 12]. Magnetic resonance imaging (MRI) is the primary imaging modality for RGNT [2, 9], typically revealing a well-circumscribed, intra-axial lesion with solid, solid-cystic, and multicystic components. This lesion exhibits hypointensity on T1-weighted images and diffusion-weighted imaging and hyperintensity on T2-weighted images with minimal or absent contrast enhancement [1, 5, 6, 10, 12]. Hemorrhage, calcification, and peripheral heterogeneous enhancement are common [1, 2, 5, 12, 15].

While the exact incidence across all published cases is not established, hemorrhage is a common occurrence in RGNT. Imaging studies suggest that hemorrhagic change may be more common than clinically appreciated [2, 6, 15–19]; however, this is a frequently overlooked feature in early-stage disease [9]. Imaging reveals susceptibility changes consistent with altered blood products and overt hemorrhagic presentation (intratumoral bleed, post-traumatic bleed, and hydrocephalus-associated bleed). The proposed mechanisms of hemorrhage include focal tumor necrosis with vessel rupture, trauma-induced intratumoral bleeding, and the presence of previous microhemorrhages or blood product deposition (including hemosiderin) on susceptibility-weighted imaging (SWI) [9, 15–19]. Importantly, hemorrhagic presentation in RGNT does not appear to correlate with malignant behavior or adverse prognosis in some case reports [6, 9, 15, 16, 19].

Intraoperative findings often reveal soft, gelatinous lesional tissue, which appears grayish pink and is mildly to moderately vascular [1, 3, 5]. Histopathological examination reveals predominantly well-demarcated lesions, although isolated foci of limited invasion may be identified [1]. According to the WHO Classification of Tumors of the Central Nervous System, 5th edition (WHO CNS5), the essential diagnostic criteria are biphasic histomorphology, characterized by both neurocytic and glial components, together with uniform neurocytes forming rosettes or perivascular pseudorosettes that express synaptophysin (a neuronal marker) [1]. Other reported histologic features include microcystic areas, rosenthal fibers, eosinophilic granular bodies, calcification, hemosiderin, microvascular hyperplasia, isolated ganglion-like cells, and absence of hypercellularity or necrosis [1, 2, 6, 9]. While histopathologic demonstration of dystrophic calcification, hemorrhage, and hemosiderin suggests susceptibility for microvascular bleeding, these features have not been consistently demonstrated.

Immunoreactivity for glial fibrillary acidic protein (GFAP) (a glial marker), S100 protein, and nuclear OLIG2 staining is present in the glial component, but negative in the rosettes and pseudorosettes. The neurocytic tumor cells may exhibit cytoplasmic expression of microtubule-associated protein 2 (MAP2), neuron-specific enolase (NSE), neuronal nucleus antigen (NeuN), and neurofilament. The Ki-67 proliferation index is typically low (range 0.35% to 3.07% in reported cases), and mitotic figures are usually absent. The tumor also shows a wild-type p53 staining pattern (helps exclude TP53-mutant diffuse gliomas) and negative IDH1 (R132H) expression (helps exclude IDH-mutant diffuse gliomas) [1, 2, 5, 6, 9].

The main pathological differential diagnosis of RGNT is pilocytic astrocytoma [1]. Other key histologic mimics include dysembryoplastic neuroepithelial tumor, oligodendroglioma, ependymoma, and central neurocytoma [3, 5, 6, 20]. As summarized in Table 1, a careful assessment of architectural patterns, neuronal differentiation, immunophenotype, and anatomic location allows for a reliable distinction of RGNT from its principal morphologic mimickers in most cases [21–26].

**Table 1:** Key features distinguishing rosette-forming glioneuronal tumor from common posterior fossa and intraventricular mimics

Tumor entity	Typical age	Typical location	Imaging features	Key diagnostic feature	Key IHC profile
Rosette-forming glioneuronal tumor (CNS WHO grade 1)	Children, adolescents, young adults	Fourth ventricle, cerebellum, brainstem	Well-circumscribed, often solid, cystic-solid, or multicystic; minimal or no enhancement	Biphasic tumor with true neuropil-centered neurocytic rosettes and glial component	GFAP + (glial component), synaptophysin + (rosettes), EMA-
Pilocytic astrocytoma (CNS WHO grade 1)	Children, young adults (first two decades of life)	Cerebellum (most common); also optic nerve and midline locations	Well-circumscribed cystic lesion with enhancing mural nodule	Biphasic astrocytic tumor with Rosenthal fibers and eosinophilic granular bodies	GFAP +, OLIG2 +, S100 +, synaptophysin −/+, NeuN-, NFP-
Dysembryoplastic neuroepithelial tumor (CNS WHO grade 1)	Children, young adults	Cerebral cortex, predilection for temporal lobe	Cortical-based, lobulated/“bubbly” appearance, sharply defined margin, no mass effect or enhancement	Floating neurons in mucin pools	Synaptophysin + (floating neurons), S100 +, NeuN + neurons, OLIG2 +, GFAP-
Oligodendroglioma, IDH-mutant and 1p/19q-codeleted (CNS WHO grade 2 or 3)	Adults (fourth and fifth decades of life)	Cerebral hemispheres (rarely observed in midline structures)	Cortical or subcortical hypodense or isodense mass lesions, calcifications common	Diffuse infiltrative growth, “fried-egg” cells (artefact seen in FFPE tissue), “chicken wire” vasculature	IDH1 p.R132H +, OLIG2 +, S100 +, GFAP +, Synaptophysin −
Ependymoma (CNS WHO grade 2 or 3)	Children and adults	Supratentorial region, posterior fossa, and spine	Heterogeneous mass, calcifications, and cysts common	Uniform small round cells; perivascular pseudorosettes, true ependymal rosettes	GFAP +, EMA + (dot-like), Synaptophysin −, OLIG2-
Central neurocytoma (CNS WHO grade 2)	Young adults (20 to 40 years)	Lateral ventricles, third ventricles	Intraventricular mass, mixed solid and cystic, frequent calcifications	Sheets of uniform round neurocytic cells	Synaptophysin +, NeuN +, GFAP − or focal

Data adapted from references [17–23]

*CNS* central nervous system, *EMA* epithelial membrane antigen, *FFPE* formalin-fixed paraffin-embedded tissue, *GFAP* glial fibrillary acid protein, *NeuN* neuronal nuclei antigen, *NFP* neurofilament protein, *OLIG2* oligodendrocyte transcription factor 2, *S100* S100 calcium-binding protein

In limited or unresolved small biopsy specimens demonstrating only a single tumor component, methylation profiling consistent with RGNT may support the diagnosis [1]. Molecularly, RGNTs are frequently associated with *FGFR1* mutations, which frequently co-occur with *PIK3CA* and/or *NF1* mutations. However, these molecular findings are considered desirable rather than mandatory when classical morphologic and immunophenotypic features are present [1]. This is particularly important in resource-limited settings (RLS), where access to molecular diagnostics and methylation profiling may be limited, necessitating reliance on integrated clinicopathologic and radiologic assessment. For example, below we present a case that illustrates the diagnostic reliability of WHO CNS5-defined essential morphologic and immunophenotypic criteria, interpreted within an appropriate clinical and radiologic context, to establish a confident diagnosis of RGNT in the absence of molecular testing.

## Management

Surgical resection is the cornerstone of therapy, with gross total resection (GTR) the primary modality, and subtotal resection (STR) as an alternative. While GTR is considered the most effective treatment option and is typically curative with uncommon recurrence, the procedure is often challenging due to the tumor’s location, which is associated with a significant risk of neurological [2, 5, 9, 10, 27]. While STR (debulking) is associated with recurrence, there appears to be no significant difference in progression between GTR and STR, with some authors postulating that aggressive surgery may not be necessary in all cases [2, 10, 27].

There is limited evidence on the efficacy of adjuvant radiotherapy or chemotherapy due to its limited use. However, there are reports of its use in isolated cases following incomplete resection, recurrence, dissemination, or in atypical cases leading to a stable tumor remnant, with no reported recurrence [2, 10, 27]. In the era of precision medicine, pharmacological treatment strategies for rosette-forming glioneuronal tumors remain an area of ongoing investigation, with isolated reports suggesting a potential role for bromocriptine in mitigating postoperative cerebellar mutism, a complication commonly associated with posterior fossa surgery [9]. Finally, emerging data suggest potential targeted therapies for tumors with drug-targetable mutations, such as erdafitinib and pemigatinib for tumors with *FGFR1* mutations, or alpelisib for tumors with *PI3K* pathway mutations [9].

## Prognosis and outcomes

RGNTs have an excellent prognosis in terms of disease-free survival, with reported 2-year disease-free survival rates exceeding 95–100% following GTR. Recurrence is uncommon (approximately 5–10%) and occurs predominantly after STR, indicating that disease control is more closely related to completeness of resection than intrinsic tumor aggressiveness [1–3, 9, 10]. Although overall survival remains excellent, extended follow-up suggests a gradual decline in disease-free survival over time, with reported 10-year disease-free survival rates approaching 50%, supporting the need for long-term surveillance [9, 14].

Functional outcomes are generally favorable, although disabling postoperative deficits have been reported in approximately half of the cases, reflecting the typical posterior fossa and brainstem-adjacent location of these tumors [1, 6]. While RGNTs are typically indolent, tumor dissemination or malignant transformation is exceptionally rare but has been documented in isolated cases, most notably with progression to high-grade astrocytoma over time [6, 9, 10]. Emerging molecular data suggest that alterations involving the *PI3K/AKT/mTOR* pathway, including *PIK3CA* mutations and rare *TERT* promoter mutations, may be associated with more aggressive behavior [9], although molecular profiling remains supportive rather than essential in routine practice [1].

## Exemplary case description

**Fig. 1:**
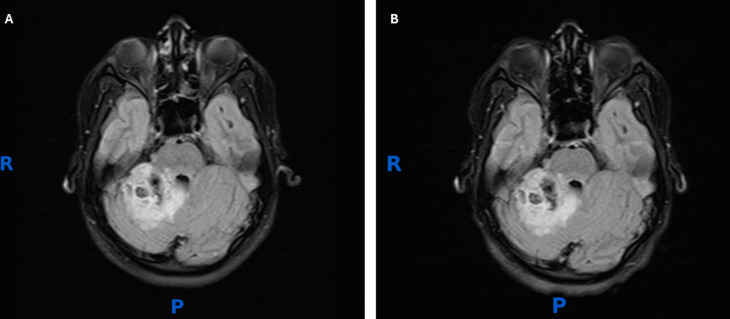
Serial non-contrast axial T2-weighted magnetic resonance images (MRI) of the posterior fossa lesion. **A** Initial MRI showing a well-circumscribed heterogeneous, solid cystic lesion (44 × 44 mm) centered on the right middle cerebellar peduncle and superior cerebellar vermis, with associated mass effect and partial effacement of the fourth ventricle; and no hydrocephalus. The lesion is hyperintense. **B** Follow-up MRI demonstrating interval stability of the right cerebellar lesion without evidence of significant interval change in size (45 × 39 × 36 mm) or characteristics, consistent with an indolent lesion. Susceptibility-weighted imaging (SWI) sequences (not shown) demonstrated a blooming artifact consistent with altered blood products within the center of the lesion

The present case is that of a 19-year-old young woman who presented in April 2022 with headache and vomiting, without focal neurological deficits. Initial CT scan demonstrated a (spontaneous) right cerebellar peduncle bleed, concerning for a cavernoma. Angiographic studies, including digital subtraction angiography (DSA), demonstrated no evidence of arteriovenous malformation or other vascular lesion. MRI scan of the brain performed 3 months later revealed a right cerebellar lesion (44 × 44 mm axial) involving the right middle cerebellar peduncle and adjacent cerebellar hemisphere, causing partial effacement of the fourth ventricle. There was no upstream hydrocephalus or herniation. The lesion was predominantly T2/FLAIR hyperintense with internal hypointense foci and showed no enhancement (Fig. 1A). Time-of-flight magnetic resonance angiography (TOF MRA) was performed and showed no arterial vascular abnormality, arguing against an underlying arteriovenous malformation or aneurysm.

A follow-up MRI of the brain (pre-contrast and post-contrast) performed 6 months later (Fig. 1B) revealed no significant interval change in the size or imaging characteristics of the right cerebellar solid-cystic mass (45 × 39 × 36 mm), which was hyperintense on T2-weighted imaging (T2W). A susceptibility-related blooming artifact consistent with the presence of altered blood products was present within the center of the lesion on T2W and SWI. The lesion was heterogeneous on T1-weighted sequences (with hypointense solid and cystic components) with no demonstrable enhancement. The lesion was centered on the brachium pontis (middle cerebellar peduncle) and superior cerebellar vermis. There was a mass effect on the pons and inferior colliculus, but no effacement of the fourth ventricle or hydrocephalus.

Despite the initial hemorrhagic presentation, the persistence of a mass-like lesion with stable configuration on serial imaging raised concern for an underlying neoplasm rather than a resolving hematoma or vascular malformation. The patient underwent elective STR (debulking) in August 2023, approximately 17 months after the initial clinical presentation. GTR was not pursued due to tumor location (middle cerebellar peduncle and superior cerebellar vermis) and the proximity to critical cerebellar outflow tracts, deep cerebellar nuclei, and brainstem structures, where aggressive resection would carry a high risk of postoperative mutism and permanent neurological morbidity. Intraoperatively, the tumor was described as soft and gelatinous, with friable, bleeding vessels.

**Fig. 2:**
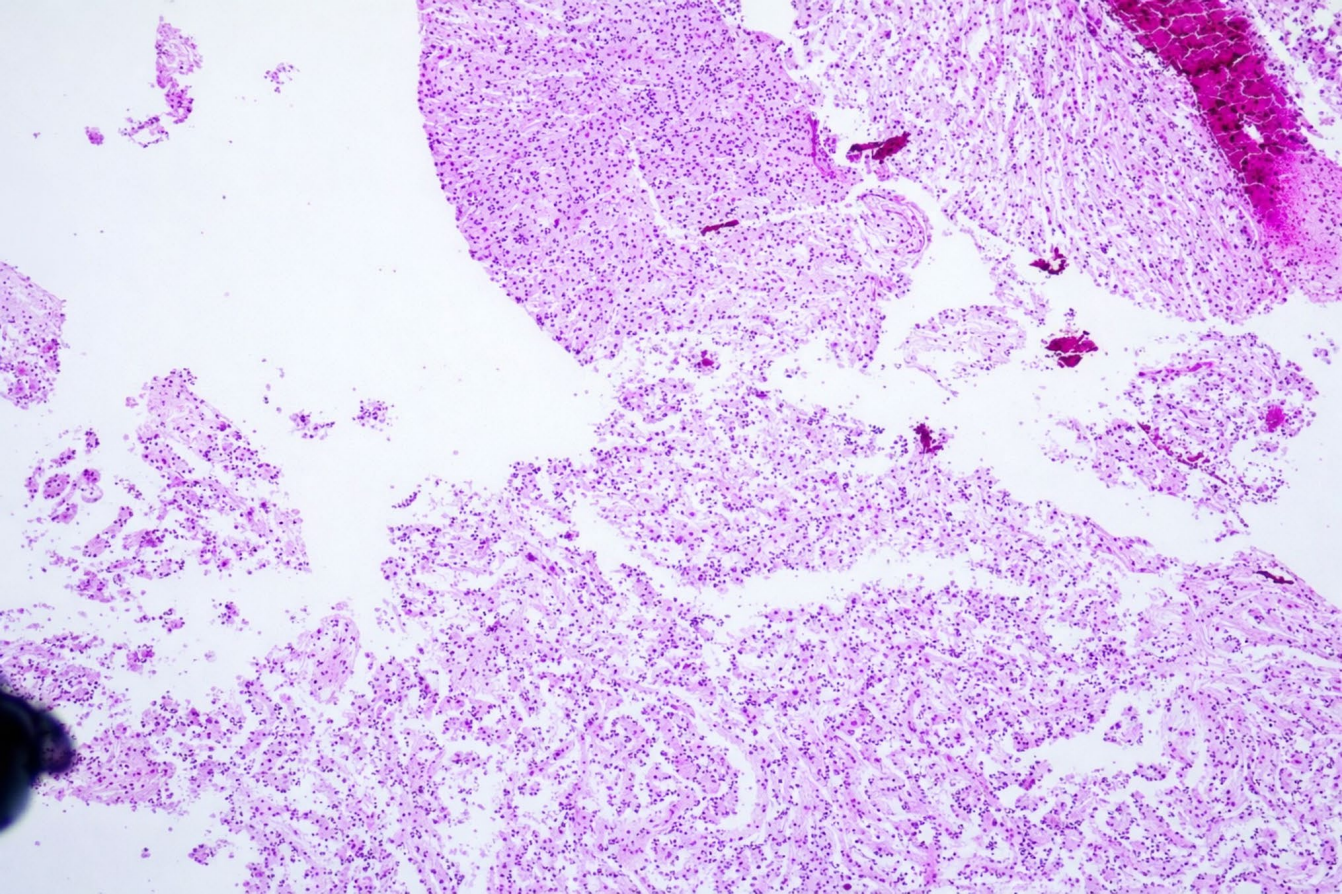

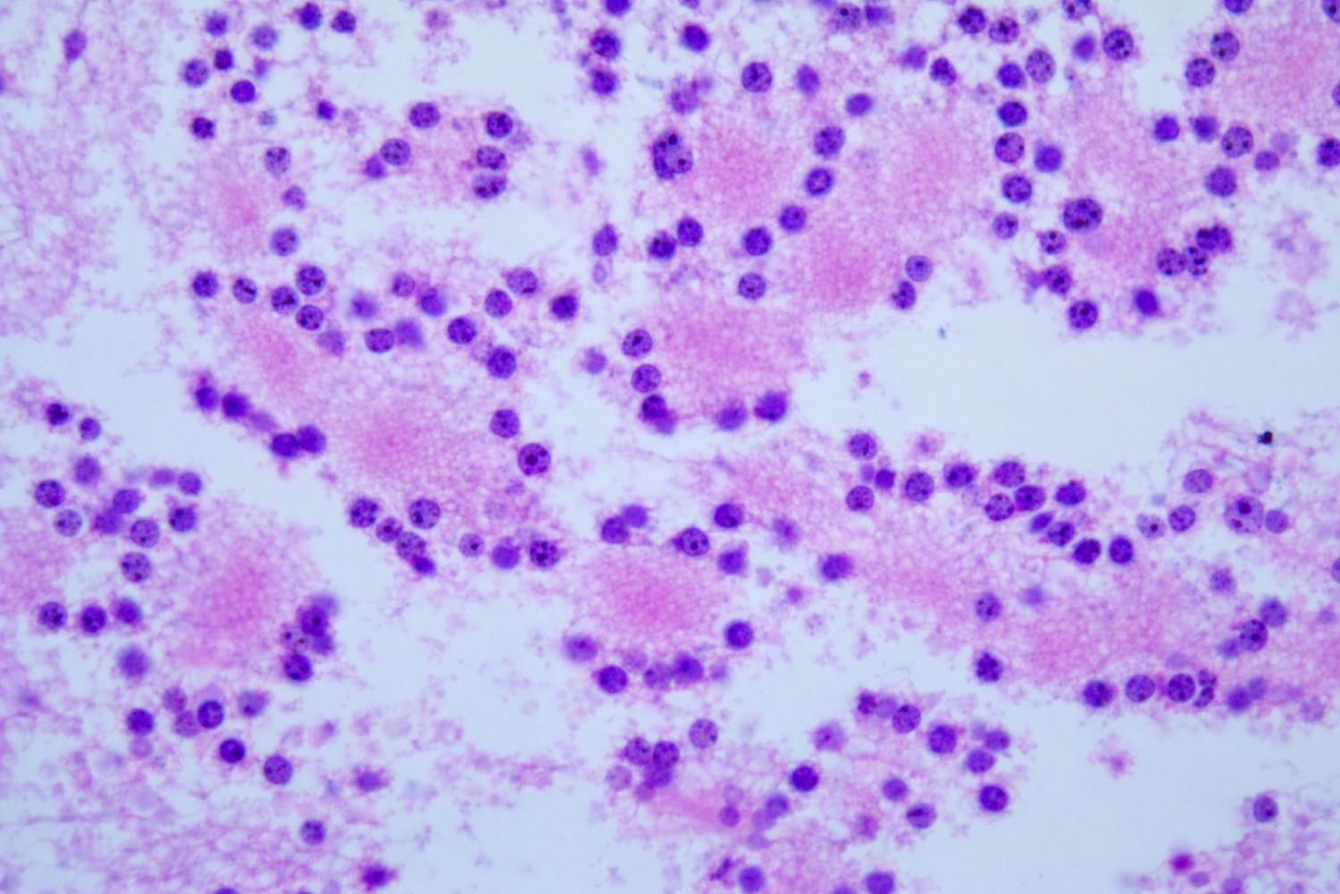

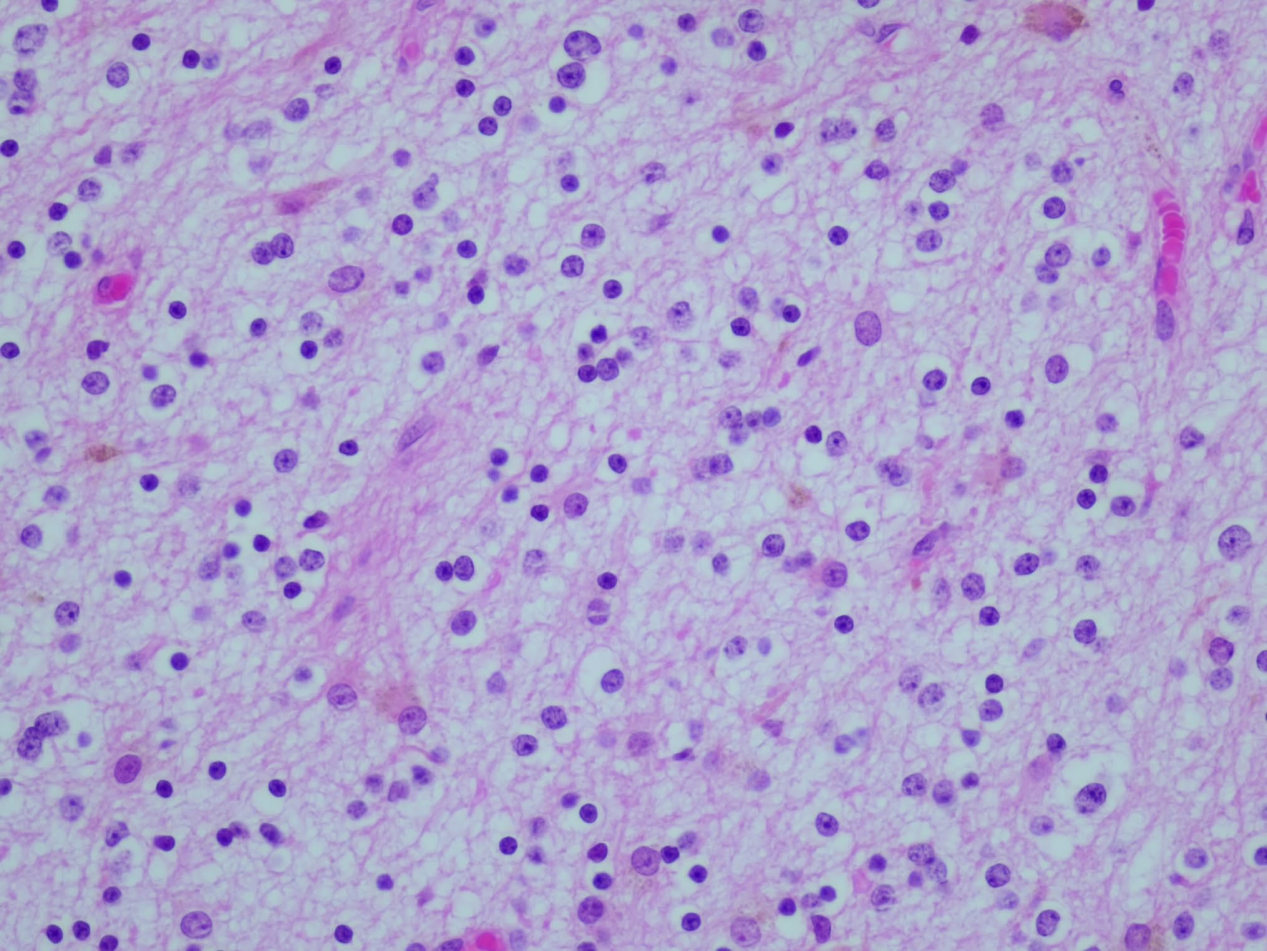
Histologic features. **a** Rosette-forming glioneuronal tumor consisting of two components: neurocytic (bottom) and astrocytic (top). **b** Neurocytic rosettes forming ring-like arrays of neurocytic tumor cell nuclei around eosinophilic neuropil cores. **c** Glial component of RGNT showing a fibrillary, piloid astrocytic background with bland cytology and low cellularity (H&E)

The surgical pathology specimen consisted of multiple tissue fragments, measuring 3–7 mm in greatest dimension. Histologic sections showed a low-grade glioneuronal neoplasm composed predominantly of a uniform population of small, round neurocytic cells with bland, centrally placed nuclei and frequent perinuclear clearing. Numerous well-formed neurocytic rosettes were present, with tumor cells radially arranged around eosinophilic neuropil-like cores. No perivascular pseudorosette-like arrangements were identified. The background was fibrillary and hypocellular, containing scattered glial cells with elongated processes imparting a piloid to oligodendroglia-like appearance. Psammomatoid calcifications were focally present. No brisk mitotic activity, microvascular proliferation, or necrosis was identified (Fig. 2a–c).

**Fig. 3:**
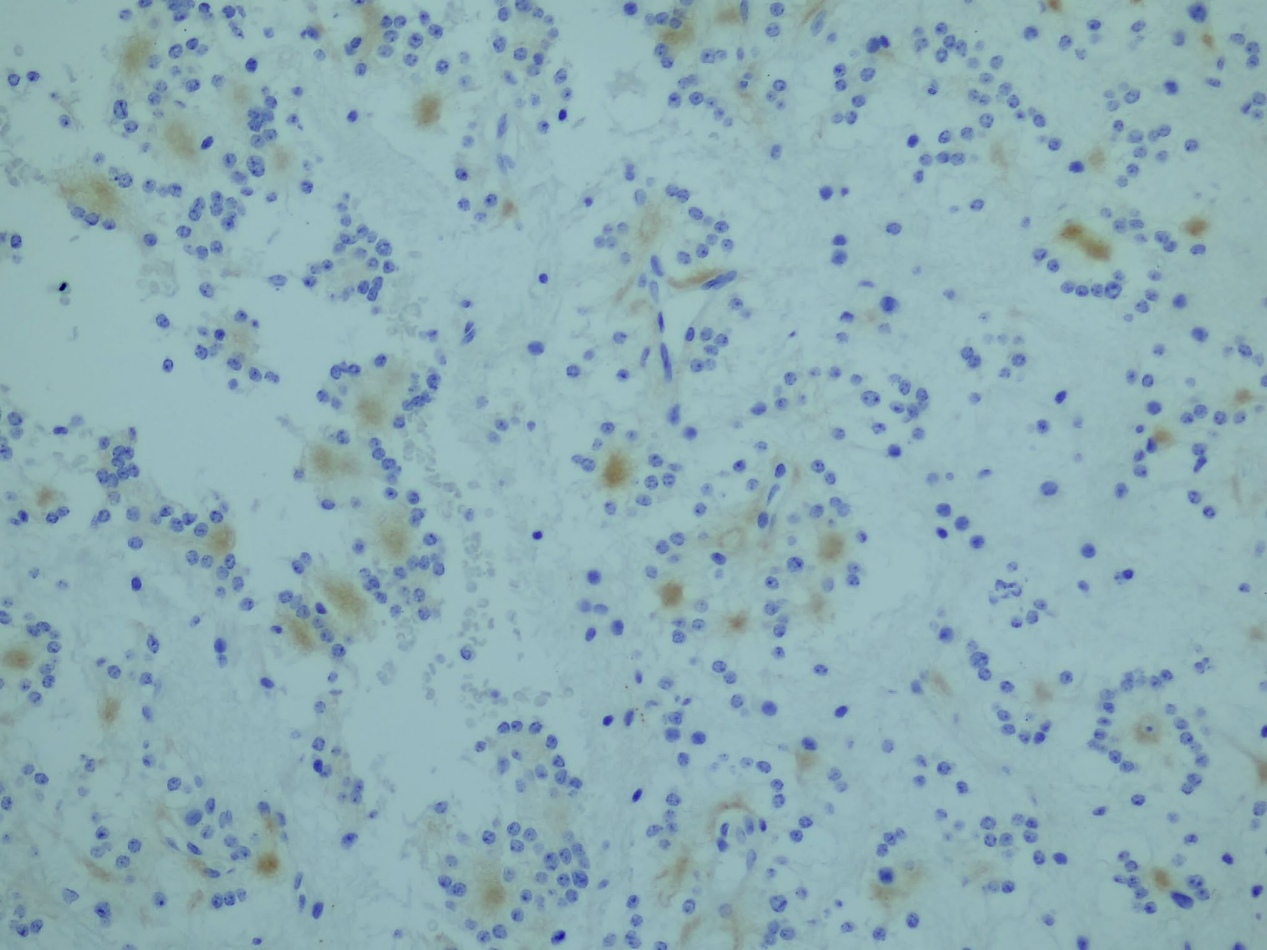

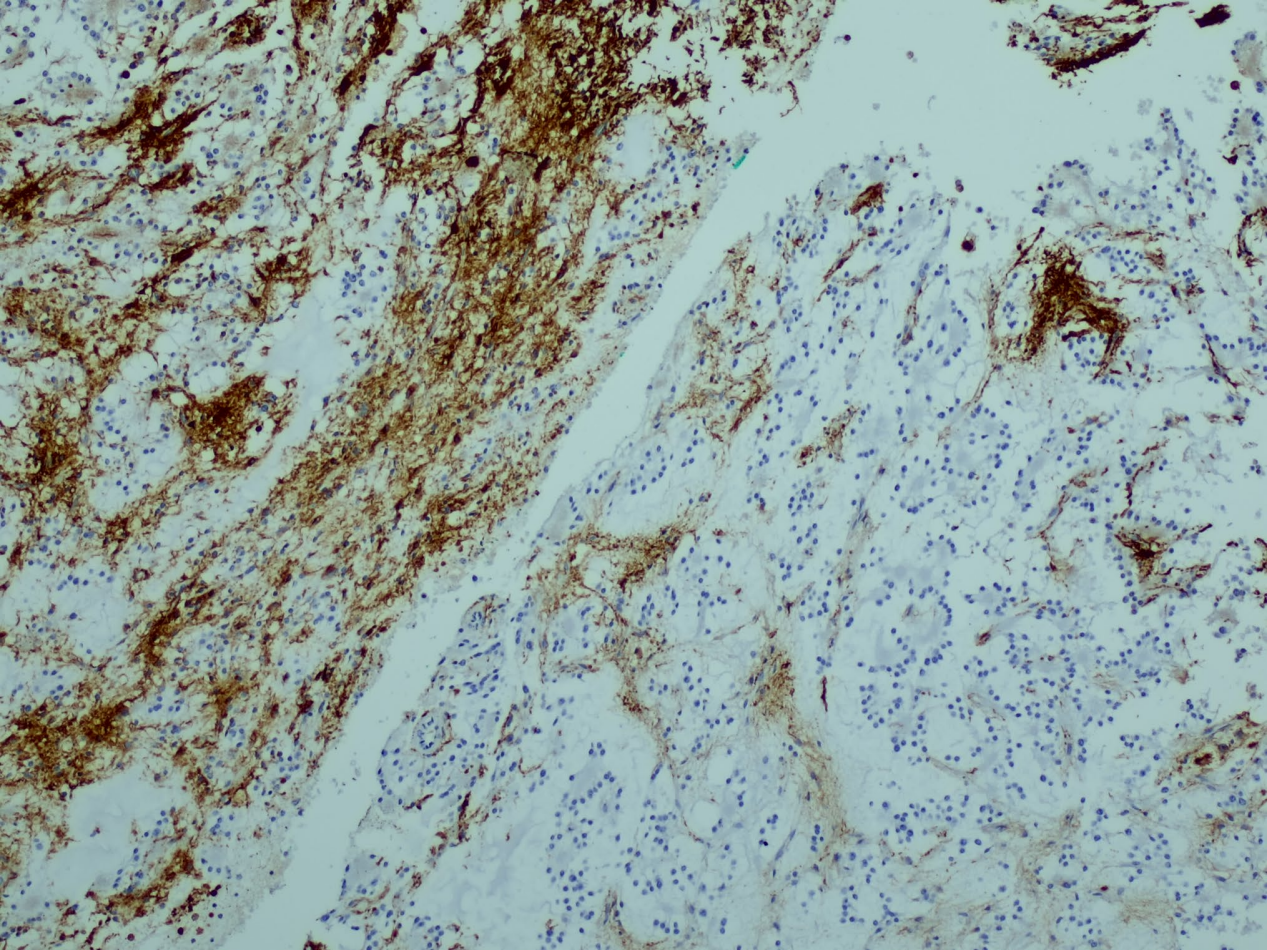

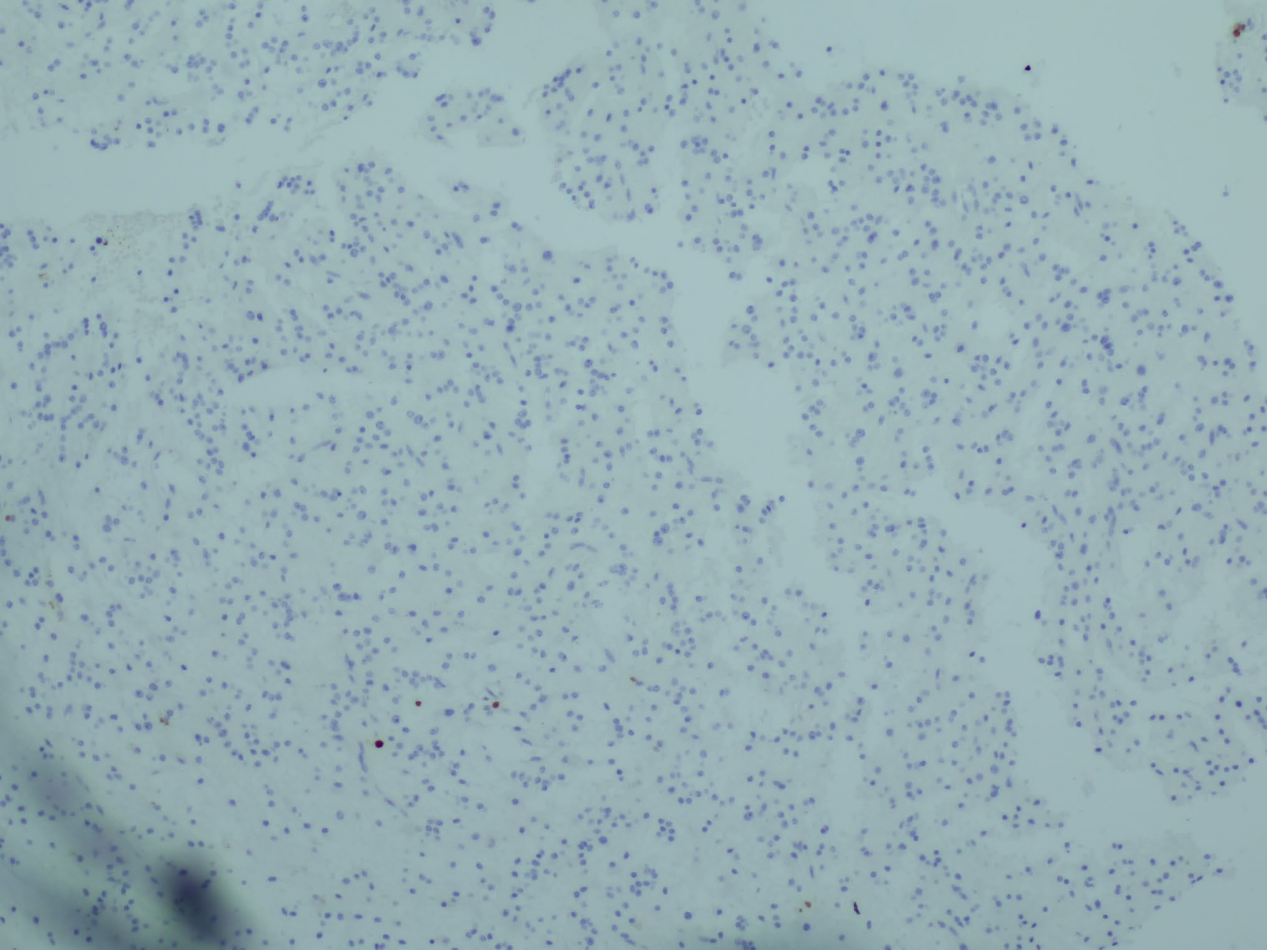
Immunohistochemical features. **a** Synaptophysin immunoreactivity of the neuropil cores of neurocytic rosettes. **b** Glial fibrillary acidic protein (GFAP) immunoreactivity is confined to the glial component and absent in rosettes and pseudorosettes. **c** Ki-67 proliferation index was < 1%

Immunohistochemistry was performed on formalin-fixed, paraffin-embedded tissue using standard protocols with appropriate controls. Synaptophysin demonstrated positivity within the neuropil cores of neurocytic rosettes, confirming neuronal differentiation. Although the staining intensity was relatively weak, the distribution was specific to rosette cores and concordant with classic RGNT morphology (Fig. 3a). GFAP immunoreactivity was confined to the glial component and absent in rosettes (Fig. 3b). IDH1 (R132H) was negative, p53 showed a wild-type staining pattern, and the Ki-67 proliferation index was < 1% (Fig. 3c), consistent with a low-grade neoplasm. Additional neuronal markers, such as NeuN, MAP2, and OLIG2, were not available in our resource-limited setting; therefore, the diagnosis was based on classic histomorphology and essential immunohistochemical features as defined by the WHO CNS5.

### Integrated diagnostic synthesis

The prolonged radiologic persistence of a non-enhancing, solid cystic posterior fossa lesion centered on the middle cerebellar peduncle, together with the intraoperative findings of a soft gelatinous tumor with bleeding vessels, supported the diagnosis of an underlying low-grade glioneuronal neoplasm rather than a resolving hematoma or vascular malformation. Histologic examination revealed a biphasic tumor composed of a neurocytic component that formed synaptophysin-positive neuropil-centered rosettes and an accompanying glial component, fulfilling the essential diagnostic criteria for a rosette-forming glioneuronal tumor, classified as a WHO CNS grade 1 tumor [1]. Although molecular testing was not performed, the concordance between radiologic features, operative findings, and classic histomorphology, together with the immunophenotype, enabled a confident integrated diagnosis.

Postoperative management consisted of clinical observation and MRI, with the aim of monitoring residual tumor stability given the indolent nature of RGNT and the absence of radiologic progression. Follow-up clinical and radiologic surveillance has demonstrated stable residual disease without progression at the last clinical visit, 24 months postoperatively. Although the patient has experienced persistent tinnitus, they have reported no new neurological deficits or decline in functional ability.

## Conclusion

Rosette-forming glioneuronal tumors are uncommon posterior fossa neoplasms with generally favorable biological behavior but potentially challenging diagnostic and surgical considerations due to their location and variable presentation. Current evidence supports a conservative, individualized management approach, prioritizing maximal safe resection while minimizing neurological morbidity. Subtotal resection may be appropriate in anatomically constrained cases, with long-term surveillance playing a central role in care.

Accurate histopathological diagnosis remains fundamental to optimal management. When the WHO CNS5 essential morphologic and immunophenotypic criteria are fulfilled, a confident diagnosis can be achieved without reliance on molecular testing, even in atypical presentations. For clinicians and pathologists, particularly in resource-limited settings, integrated clinicoradiologic and histopathologic evaluation continues to provide a reliable and practical framework for the diagnosis and management of RGNT.

## Data Availability

No datasets were generated or analysed during the current study.
